# Restrictions in Cell Cycle Progression of Adult Vestibular Supporting Cells in Response to Ectopic Cyclin D1 Expression

**DOI:** 10.1371/journal.pone.0027360

**Published:** 2011-11-02

**Authors:** Heidi Loponen, Jukka Ylikoski, Jeffrey H. Albrecht, Ulla Pirvola

**Affiliations:** 1 Institute of Biotechnology, University of Helsinki, Helsinki, Finland; 2 Helsinki Ear Institute, Helsinki, Finland; 3 Division of Gastroenterology, Hennepin County Medical Center, Minneapolis, Minnesota, United States of America; University of Chicago, United States of America

## Abstract

Sensory hair cells and supporting cells of the mammalian inner ear are quiescent cells, which do not regenerate. In contrast, non-mammalian supporting cells have the ability to re-enter the cell cycle and produce replacement hair cells. Earlier studies have demonstrated cyclin D1 expression in the developing mouse supporting cells and its downregulation along maturation. In explant cultures of the mouse utricle, we have here focused on the cell cycle control mechanisms and proliferative potential of adult supporting cells. These cells were forced into the cell cycle through adenoviral-mediated cyclin D1 overexpression. Ectopic cyclin D1 triggered robust cell cycle re-entry of supporting cells, accompanied by changes in p27^Kip1^ and p21^Cip1^ expressions. Main part of cell cycle reactivated supporting cells were DNA damaged and arrested at the G2/M boundary. Only small numbers of mitotic supporting cells and rare cells with signs of two successive replications were found. Ectopic cyclin D1-triggered cell cycle reactivation did not lead to hyperplasia of the sensory epithelium. In addition, a part of ectopic cyclin D1 was sequestered in the cytoplasm, reflecting its ineffective nuclear import. Combined, our data reveal intrinsic barriers that limit proliferative capacity of utricular supporting cells.

## Introduction

The very limited capacity of the inner ear sensory epithelial cells to proliferate either naturally or following traumas is a critical barrier for hair cell regeneration in mammals. Experimental manipulation of the retinoblastoma tumor suppressor protein (pRb) pathway in the inner ear has demonstrated a death-prone phenotype of cell cycle reactivated hair cells [1]–[6], similar to many other types of postmitotic cells, such as neurons and myotubes [7], [8]. Unscheduled DNA replication and associated DNA damage cause acute death of auditory hair cells. Vestibular hair cells forced into S phase can resist acute death, but also their DNA is damaged and they are arrested in the cell cycle, with an apparent death-prone ultimate fate [6]. These prior data revealing barriers in the proliferative response of hair cells imply that the postmitotic state is critical for the life-long survival of these cells and that the maintenance of quiescence is an active process, regulated by several cell cycle molecules.

The other main cell type of the inner ear sensory epithelia is supporting cells that, in mammals, are postmitotic, similar to hair cells. In the lesioned mammalian inner ear, supporting cells have a glial cell-like function; they close the lesion and form a scar that replaces lost hair cells. In contrast to mammals, non-mammalian species possess a natural regenerative capacity in their inner ears; their supporting cells can divide and transdifferentiate into new hair cells, leading to restoration of hearing and balance functions [9], [10]. Because of this remarkably different capacity for sensory cell regeneration, there is considerable interest in understanding the molecular mechanisms underlying the restrictions in proliferation in mammalian supporting cells.

Mutant mouse models have shown that pRb, the prototypical member of the pocket protein family, and p27^Kip1^, a member of the Cip/Kip family of cyclin-dependent kinase inhibitors (CKIs), regulate the maintenance of the postmitotic state of cochlear and vestibular supporting cells [11]–[13]. In addition, p27^Kip1^ is a critical regulator of cell cycle exit of embryonic precursor cells common for hair cell and supporting cell lineages [14], [15]. Vestibular supporting cells can synthesize DNA in response to exogenous mitogens neonatally, but this capacity is lost at later stages [16]–[21]. Also dissociated and purified cochlear supporting cells can re-enter S phase neonatally, but not anymore thereafter. This age-dependent plasticity for cycle reactivation was linked with differential capacity of neonatal versus older cochlear supporting cells to downregulate p27^Kip1^ [22]. In addition, cyclin D1 (cD1), which is a positive cell cycle regulator critical in G1/S transition, has been shown to be expressed in a dynamic pattern in the inner ear supporting cells. Specifically, cD1 downregulation was shown to closely parallel with the early postnatal stage when supporting cells show a steep decline in mitogen responsiveness [23].

It has been shown in neonatal cochlear explant cultures that silencing of *p27^Kip1^* by siRNA stimulates supporting cell's DNA replication, as detected by bromodeoxyuridine (BrdU) incorporation, and that these cells can progress into mitosis [11]. Another study [12] used inducible deletion of *Rb in vivo* and showed the capacity of neonatal cochlear supporting cells to re-enter the cell cycle and progress through karyokinesis and cytokinesis. It was also suggested that these cells can proceed through multiple rounds of divisions. In both of these studies, unscheduled cell proliferation was linked to apoptosis, either directly or indirectly. Neonatal postnatal day 4 to 6; P4 to P6) cochlear supporting cells used in those studies are still immature. Two other recent *in vivo* studies have investigated the proliferative response of adult, fully differentiated supporting cells using BrdU labeling as a read out of proliferative activity. Inducible inactivation of *Rb* did not at all trigger cell cycle reactivation of adult cochlear or vestibular supporting cells [14], in contrast to inducible *p27^Kip1^* deletion [13]. This latter study found that the extent of DNA synthesis is substantially reduced in adult cochlear and vestibular supporting cells deficient for *p27^Kip1^* as compared to their neonatal (P7) counterparts.

Based on our earlier data showing the absence of cD1 expression in the majority of supporting cells of the adult mouse inner ear [23], we have here asked if adenovirus-mediated cD1 overexpression can override this barrier and, specifically, if adult supporting cells can progress through the cell cycle and divide. We addressed this question in organotypic cultures of the utricle, one of the vestibular organs. Adult supporting cells form the platform for the development of strategies to replace lost sensory hair cells, and stimulation of cell proliferation is a central part of this development. Current results are likely to contribute to the understanding of the mechanisms that limit proliferative potential of mammalian supporting cells.

## Results

**Figure 1:**
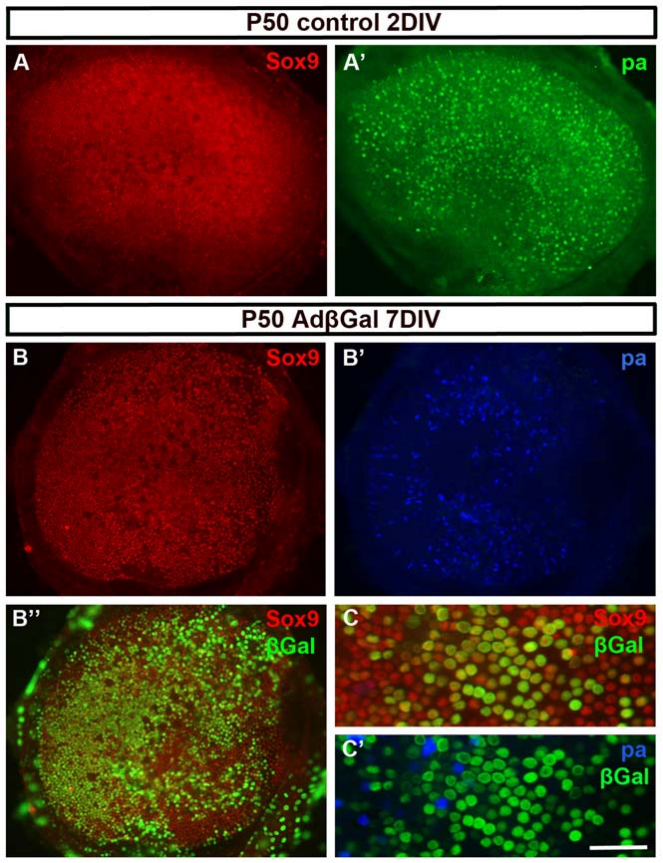
Adenoviruses infect adult utricular supporting cells *in vitro*. (A, A') P50 explants maintained for 2 DIV show a homogenous layer of Sox9+ supporting cells (A) and parvalbumin+ hair cells (A'), based on double-immunofluorescence. (B-B″) At 7 DIV, triple-immunofluorescence reveals that the homogenous layer of Sox9+ supporting cells is maintained, but there is a loss of a large part of parvalbumin+ hair cells. β-galactosidase staining shows that supporting cell infectivity is more than 50% (B″). (C,C'). This higher magnification view from an explant triple-stained for Sox9, β-galactosidase and parvalbumin shows transgene expression in a large part of supporting cells, in contrast to remaining hair cells. Abbreviations: pa, parvalbumin; βGal, β-galactosidase. Scale bar, shown in C': A–B″, 140 µm; C,C', 60 µm.

The transcription factor Sox9 has earlier been localized to supporting cells of the late-embryonic inner ear sensory epithelia [25]. We found that this cell type-specific expression persisted throughout postnatal life. Untreated utricular explants and explants infected with an adenovirus encoding β-galactosidase (AdβGal) were used as control specimens. They showed Sox9 expression in all supporting cells, i.e. both in the striolar (central) and extra-striolar part of the utricular sensory epithelium. Based on these findings, we used Sox9 as a supporting cells marker in the current study. Utricles prepared from P50 mice and maintained for 2 to 12 days *in vitro* (DIV) showed a homogenous layer of Sox9+ supporting cells (Fig. 1A,B). After 2 DIV, control utricles showed a normal complement of hair cells, as evidenced by immunostaining for the hair cell markers myosin VIIa (data not shown) and parvalbumin (Fig. 1A′). A large part of hair cells were lost by 7 DIV, the extent of this loss being variable between explants (Fig. 1B′). For comparison, under the same culture conditions and time, utricles from P9 mice showed a largely intact complement of hair cells, based on staining these early postnatal explants with the hair cell-specific markers (data not shown). Consistent with these data, prior studies have shown the vulnerabilty of mature utricular hair cells maintained *in vitro* for more than a few days [26]. Importantly, hair cell loss did not affect supporting cell's quiescence, as evidenced in untreated and control explants by the absence of cells positive for the proliferation marker Ki-67. In addition, hair cell loss did not impair supporting cell's survival, based on the lack of cells with DNA fragmentation (ApopTag Kit) that marks apoptotic death (data not shown). Based on these results and on the fact that the platform for therapeutic approaches is a sensory epithelium with hair cell loss, we considered that our adult utricular explants represent a good model for a lesioned sensory epithelium.

### Adenoviruses efficiently infect postnatal utricular supporting cells in vitro

For gene transfer, we used adenoviruses of serotype 5 harbouring the CMV promoter. Tropism of these viruses towards hair cells and supporting cells changes dramatically during the early postnatal maturation period. Prior *in vitro* studies have shown that hair cells are the main cell type in the inner ear sensory epithelia infected by adenoviruses at late-embryogenesis and at birth and that hair cells become refractory to these viruses gradually during the first and second postnatal weeks [23], [27]. We found that supporting cells have an opposite profile of tropism. The amount of AdβGal-infected utricular supporting cells increased during the early postnatal life (data not shown), leading to infectivity of more than 50% of these cells in P50 explants (Fig. 1B–C). In these adult cultures, remaining hair cells did not show transgene expression (Fig. 1C'). We conclude that adenoviruses efficiently and specifically transfer genes into adult supporting cells *in vitro*.

### Ectopically expressed cD1 triggers robust cell cycle re-entry of adult supporting cells

**Figure 2:**
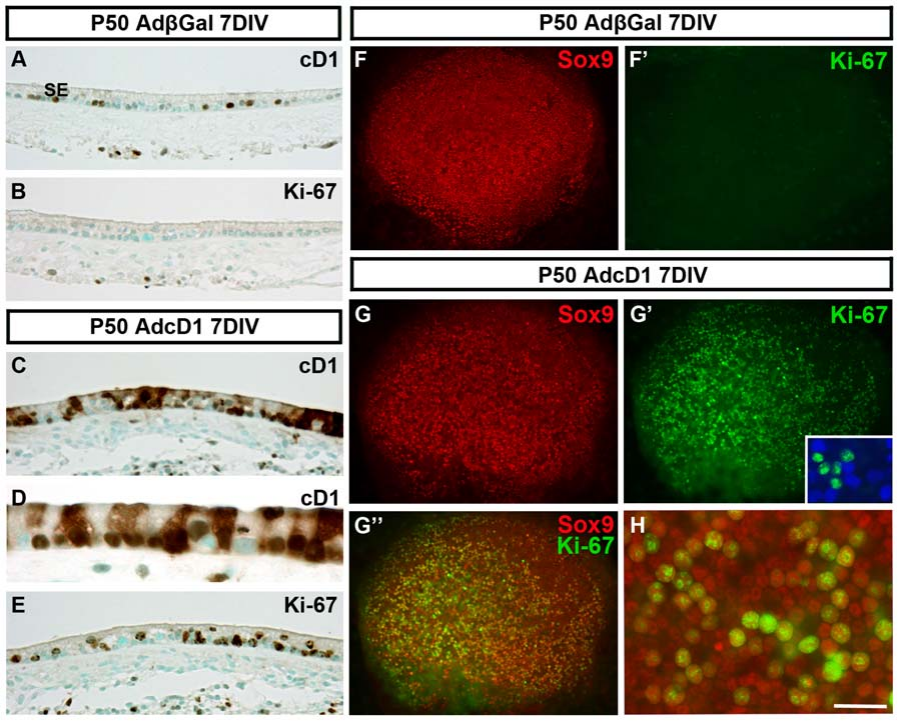
Cell cycle reactivation of adult utricular supporting cell in response to ectopic cD1 expression. (A) A histological section prepared from an AdβGal-infected P50 explant at 7 DIV shows endogenous cD1 expression in a few supporting cells. (B) An adjacent section stained with the Ki-67 antibody shows absence of cell proliferation in the sensory epithelium. (C) AdcD1-infected utricles show strong transgene expression. (D) This high magnification view shows ectopic cD1 expression both in the nuclei and cytoplasm of supporting cells. (E) An adjacent section displays high numbers of supporting cells positive for Ki-67. (F,F') Double-staining of an explant transduced by AdβGal and maintained for 7 DIV shows a homogenous layer of Sox9+ supporting cells (F) and absence of Ki-67 immunoreactivity (F'). (G–G″) Triple-labeling shows high numbers of Ki-67+/Sox9+ supporting cells in AdcD1-infected utricles at 7 DIV. Inset in G' shows that remaining parvalbumin+ hair cells do not express Ki-67. (H) This higher magnification view shows Ki-67 induction in a large part of supporting cells of AdcD1-infected explants. Abbreviations: SE, sensory epithelium; pa, parvalbumin; βGal, β-galactosidase; cD1, cyclin D1. Scale bar, shown in H: A–C,E, 70 µm; D, 30 µm; F–G″, 140 µm; H, 30 µm.

We studied the outcome of Sox9+ utricular supporting cells forced into the cell cycle. It has been earlier shown that endogenous cD1 is broadly expressed in this cell population during the first postnatal week. Thereafter cD1 expression is steeply downregulated, so that only a small part of supporting cells is positive at P9 and even less at adulthood [23]. In the present study, these prior data were confirmed in histological sections prepared from AdβGal-infected control explants (Fig. 2A). In addition, by staining adjacent sections with the Ki-67 antibody that marks the G1, S, G2 and M phases of the cell cycle, we confirmed the absence of cell proliferation in the control sensory epithelia (Fig. 2B). AdβGal-infected utricles showed a normal distribution of supporting cell nuclei in the basal half of the epithelium, next to the basement membrane (Fig. 2A,B). Transduction of P50 utricles by adenoviruses encoding cD1 (AdcD1) triggered transgene expression in supporting cells, as shown at 7 DIV (Fig. 2C). Ectopic cD1 was expressed in the supporting cell nuclei, but also in their cytoplasm. Cytoplasmic staining was very strong in a part of supporting cells (Fig. 2C,D). P50 explants transduced by AdcD1 comprised high numbers of proliferating supporting cells, a result that is clearly seen both in sections (Fig. 2E) and whole mount views (Fig. 2F–H). In AdcD1-infected explants, several Ki-67+ nuclei had translocated toward the luminal surface (Fig. 2E). Quantification performed in whole mounts at 7 DIV revealed that the amount of Sox9+/Ki-67+ supporting cells averaged 26.4%±3.5 (n = 7 explants, a total of 11276 supporting cells counted). Preserved hair cells (parvalbumin+) were invariably negative for Ki-67, in agreement with the refractoriness of mature hair cells to adenoviruses (Fig. 2G', inset). However, despite robust cell cycle activity, only a part of AdcD1-infected supporting cells re-entered the cell cycle, a result that is readily seen when comparing adjacent sections stained with the cD1 and Ki-67 antibodies (Fig. 2C, E). It is also evident when comparing the whole mount view showing transduction efficiency in an AdβGal-infected explant (Fig. 1B″) to the picture showing the extent of Ki-67 staining in an AdcD1-infected explant (Fig. 2G″). In whole mounts, due to strong cytoplasmic accumulation of ectopic cD1, double-staining for cD1 and Ki-67 was uninformative in demonstrating the extent of colocalization. Taken together, ectopically expressed cD1 triggers widespread cell cycle re-entry of adult utricular supporting cells.

### Opposite changes in p27^Kip1^ and p21^Cip1^ expressions in response to ectopic cD1

**Figure 3:**
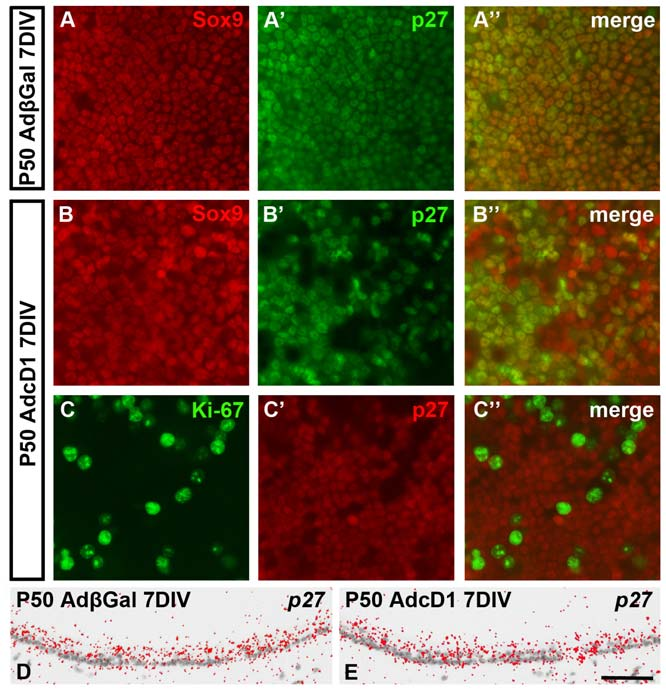
Changes in p27^Kip1^ expression in supporting cells in response to ectopic cD1 expression. (A–A″) At 7 DIV, AdβGal-infected explants show homogenous p27^Kip1^ expression in supporting cells. Sox9 is a supporting cell marker. (B–B″) In AdcD1-infected utricles, p27^Kip1^ expression is suppressed in a part of supporting cells. (C–C″) Double-staining reveals that Ki-67+ cells are negative for p27^ Kip1^. (D,E) *In situ* hybridization shows comparable *p27^Kip1^* mRNA levels in AdβGal- and AdcD1-infected explants. Scale bar, shown in E: A–C″, 40 µm; D,E, 70 µm.

To penetrate into the mechanisms behind AdcD1-triggered cell cycle reactivation, we studied the expression of p27^Kip1^ and p21^Cip1^, which are critical inhibitors of cell cycle reactivation. It has been shown that p27^Kip1^ is moderately and p21^Cip1^ very weakly expressed in postnatal utricular supporting cells [2], [23]. At 7 DIV, double-labeling of AdcD1-infected P50 explants with antibodies against p27^Kip1^and Sox9 revealed marked p27^Kip1^ suppression in supporting cells. p27^Kip1^ levels were unchanged in AdβGal-infected explants (Fig. 3A–B″). Importantly, p27^Kip1^ suppression was confined to the Ki-67+ population of supporting cells (Fig. 3C–C″). No changes were seen in *p27^Kip1^* mRNA expression, as assessed by *in situ* hybridization in paraffin sections (Fig 3D,E), indicating that AdcD1-triggered cell cycle reactivation is associated with p27^Kip1^ protein degradation. Together, these results suggest that p27^Kip1^ suppression facilitates cell cycle re-entry of supporting cells with ectopically expressed cD1.

**Figure 4:**
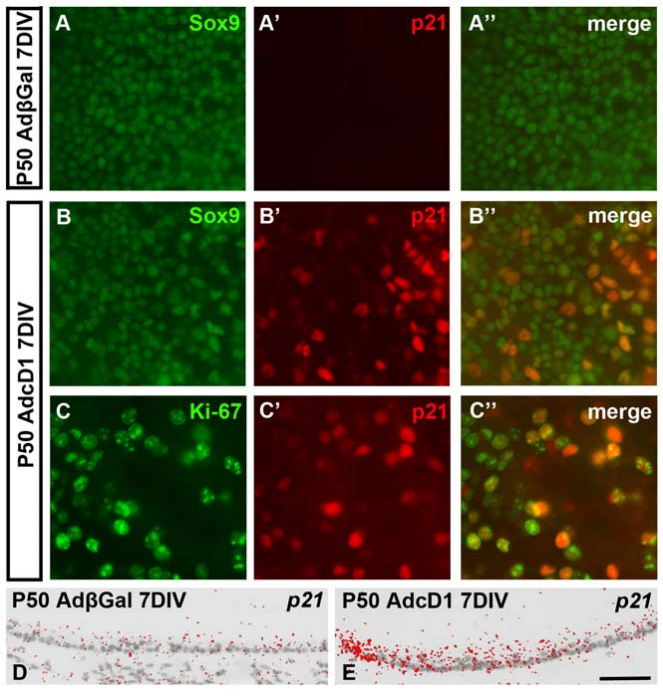
Changes in p21^Cip1^ expression in supporting cells in response to ectopic cD1 expression. (A–A″) In AdβGal-infected utricles at 7 DIV, p21^Cip1^ is absent supporting cells. Sox9 is a supporting cell marker. (B–B″) In contrast, AdcD1-infected explants at 7 DIV display strong of p21^Cip1^ upregulation. (C–C″) Double-staining shows colocalization of p21^Cip1^ and Ki-67 in suppporting cells. (D,E) *In situ* hybridization shows *p21^Cip1^* upregulation in AdcD1-, but not AdβGal-infected explants. Scale bar, shown in E: A–C″, 40 µm; D,E, 70 µm.

Also p21^Cip1^ levels were markedly altered upon cell cycle reactivation, but in opposite direction as compared to p27^Kip1^, as assessed in P50 explants at 7 DIV. Double-labeling with p21^Cip1^ and Sox9 antibodies showed strong p21^Cip1^ upregulation in supporting cells of AdcD1-, but not of AdβGal-infected explants (Fig. 4A–B″). Specifically, p21^Cip1^ induction was linked to the supporting cell population positive for Ki-67 (Fig. 4C–C″). This induction was also readily seen at the mRNA level, based on *in situ* hybridization performed on paraffin sections (Fig. 4D,E). Based on triple-labeling for p21^Cip1^, the S-phase-marker 5-ethynyl-2′-deoxyuridine (EdU) and Sox9, we confirmed that p21^Cip1^ was upregulated in supporting cells already during DNA synthesis (data not shown). To evaluate the possible anti-proliferative effect of this upregulation, explants from *p21^Cip1^* knock out mice were analyzed. Prior studies have demonstrated no developmental or phenotypic abnormalities in the inner ears of these mutant animals [2], [4]. In agreement, untreated and AdβGal-infected explants from *p21* ^Kip1^ -/- mice lacked Ki-67+ supporting cells. Mutant specimens transduced by AdcD1 comprised high numbers of Ki-67+ supporting cells (figs. not shown). This proliferative activity was comparable to that seen in AdcD1-infected wild type utricles (26.4%), based on quantification showing that 24.8%±3.2 of supporting cells in the mutant explants at 7 DIV were double-positive for Sox9 and Ki-67 (n = 4 explants, a total of 8343 supporting cells counted). Of interest, similar to wild type utricles, p27^Kip1^ expression was suppressed in Ki-67+ supporting cells of *p21^Cip1^* -/- explants (data not shown). Together, these data suggest that, despite prominent upregulation, p21^Cip1^ does not have a major role in limiting S phase initiation of AdcD1-infected supporting cells.

### Cell cycle progression is inefficient in adult supporting cells with ectopic cD1 expression

**Figure 5:**
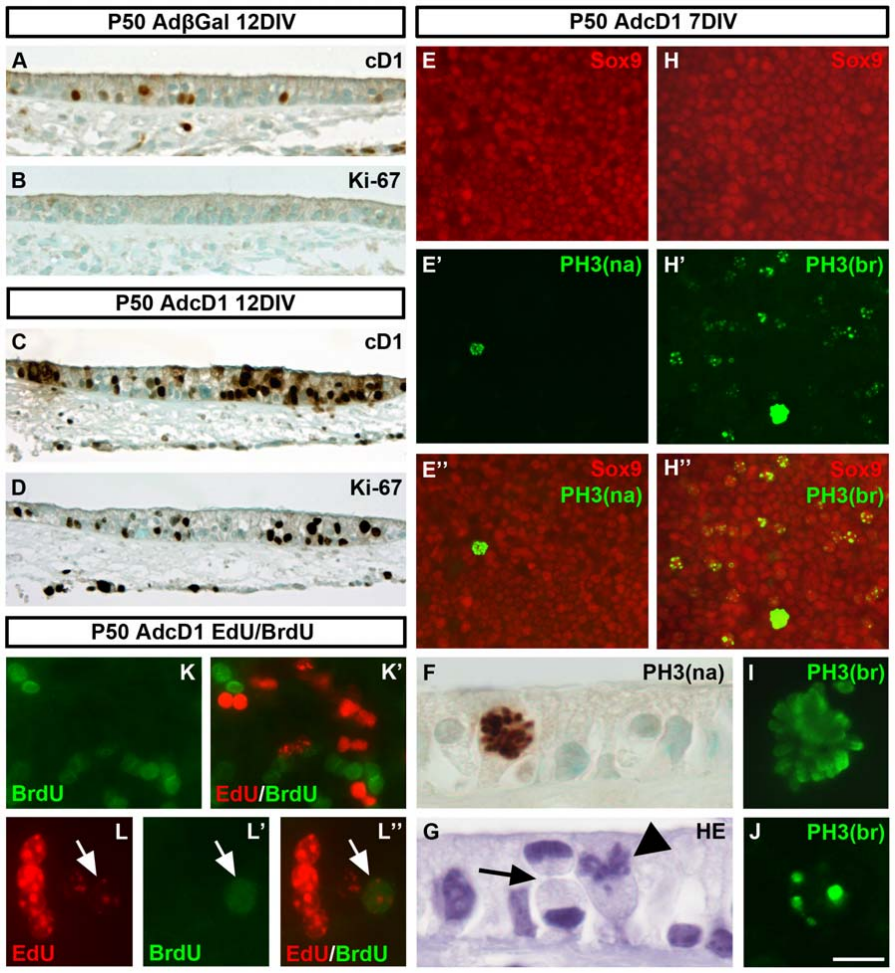
Cell cycle progression of adult utricular supporting cells forced into the cell cycle. (A,B) Adjacent histological sections prepared from AdβGal-infected explants at 12 DIV show the presence of a small number of supporting cells with endogenous cD1 expression (A) and the absence of Ki-67 immunoreactivity (B). (C) The sensory epithelium of AdcD1-infected explants at 12 DIV lacks hyperplasia, despite maintained ectopic cD1 expression. Similar to earlier postinfection time point (7 DIV), ectopic cD1 is expressed both in the nucleus and cytoplasm. (D) As shown in an adjacent section, Ki-67 expression is maintained in supporting cells of AdcD1-infected explants. (E–E″) At 7 DIV, whole mount specimens transduced by AdcD1 and stained with the “PH3-Ser10 narrow antibody” (see text) contain a small number of mitotic supporting cells. (F) A high magnification view of a histological section stained with the “PH3-Ser10 narrow antibody” shows one supporting cell undergoing prophase. (G) A hematoxylin-stained section showing one supporting cell undergoing metaphase (arrowhead) and an adjacent, rarely seen supporting cell in cytokinesis (arrow). (H–H″) At 7 DIV, whole mount specimens transduced by AdcD1 and stained with the “PH3-Ser10 broad antibody” (see text) comprise small numbers of supporting cells with prominent chromatin condensation typical to mitotic cells. In addition, this antibody labels several supporting cells with weak chromatin condensation typical to the late G2 phase. (I,J) High magnification views of supporting cells in AdcD1-infected explants stained with the“PH3-Ser10 broad antibody”. The labeling pattern readily differs between mitotic (I) and late G2 (J) cells. (K–L″) Two examples (K,K' and L–L″) of AdcD1-infected explants pulsed with the thymidine analogs EdU and BrdU (see text for the pulsing regimen). Several nuclei positive for one of the analogs can be seen (K,K'). Only rare supporting cells positive for both analogs were found (L–L″, arrows). Abbreviations: cD1, cyclin D1; PH3(na), phosphorylated histone H3-Ser10 narrow; PH3(br), phosphorylated histone H3-Ser10 broad; HE, hematoxylin; EdU, 5-ethynyl-2′-deoxyuridine; BrdU, bromodeoxyuridine. Scale bar, shown in J: A–D, 50 µm; E–E″, H–G″, 40 µm; F,G, 15 µm; I,J, 10 µm; K–L″, 20 µm.

We continued to investigate whether the widespread cell cycle re-entry of AdcD1-infected supporting cells in P50 explants leads to cellular regrowth. Paraffin sections (Fig. 5A–D) and plastic-embedded semithin sections (data not shown) were prepared from AdβGal- and AdcD1-infected explants at 12 DIV. Comparative analysis did not show signs of hyperplasia of the sensory epithelium of explants transduced by AdcD1, arguing against multiple rounds of supporting cell divisions. Immunohistochemistry on paraffin sections showed maintained cD1 overexpression and Ki-67 expression (Fig. 5A–D). We next linked these results to mitotic activity by using an antibody against phosphorylated serine 10 on histone H3 that reacts with the prominent chromatin condensation of mitotic cells (“PH3-Ser10 narrow antibody”). We did not find any mitotic supporting cells at 3 DIV and only a few of these cells were found at 12 DIV (data not shown). At 7 DIV, we detected 4.4±0.5 mitotic supporting cells per AdcD1-infected explant (n = 8 explants) (Fig. 5E–E″). This value is 0.6% of the amount of Ki-67+ supporting cells. Most mitotic supporting cells were undergoing prophase or metaphase (Fig. 5F), rather than anaphase or telophase. To detect cytokinesis, the terminal phase of the cell cycle, whole mount specimens were immunolabeled for Aurora B kinase, a component of the contractile ring at the site of cytoplasmic separation [28]. As assessed at 7 DIV, we did not find Aurora B reactivity in the utricular sensory epithelium (n = 5 explants) (data not shown). We confirmed in hematoxylin-stained paraffin sections that cytokinesis was a rare event. Fig. 5G shows the single supporting cell undergoing late mitosis that was found in sectioned material (n = 8 explants). Control specimens did not show PH3+ supporting cells, consistent with the lack of Ki-67 reactivity in these explants. Combined, these data suggest that mitotic progression is rare event in supporting cells with cD1 overexpression, consistent with the lack of hyperplasia of AdcD1-infected utricles.

We then penetrated into the mechanisms that limit cellular regrowth in AdcD1-infected P50 utricles. In addition to the “PH3-Ser10 narrow antibody” (Fig. 5E-E″), we used another PH3-Ser10 antibody that similarly labels the prominent mitotic profiles, but that also labels the weak chromatin condensation typical to the late G2 phase and G2/M transition (“PH3-Ser10 broad antibody”) [29], [30]. At 7 DIV, this antibody showed widespread labeling within the supporting cell population (Fig. 5H-H″). The weak and dotted labeling seen in most cells was clearly different from the strong signal seen in the few cells undergoing mitosis (Fig. 5I, J). Quantification showed that 11.5%±0.4 of supporting cells in AdcD1-infected explants reacted with the “PH3-Ser10 broad antibody” and showed dotted labeling pattern (a total of 8679 supporting cells counted in 7 explants). This value is 43.5% of the percentage of Ki-67+ supporting cells. As the comparable value of mitotic cells is 0.6%, we conclude that the majority of utricular supporting cells transduced by AdcD1 are arrested at the G2/M boundary.

### Rare adult supporting cells with ectopic cD1 expression have proliferative potential

To determine whether supporting cells can progress through more than one round of S phase, we pulsed AdcD1-infected P50 utricles with EdU and BrdU, two thymidine analogs that react with cells undergoing DNA replication. We first confirmed in single-pulse experiments that the BrdU antibody used did not cross-react with the EdU analog (data not shown). EdU was pulsed between days 3 and 4, and BrdU between days 7 and 8. Specimens were fixed at 9 DIV. Explants comprised several supporting cells positive for either BrdU or EdU (Fig. 5K–L″). Replication activity was higher during the early versus later pulsing period. We found EdU+/BrdU+ supporting cells, but they were rare and were seen only in a part of explants, usually only one or two per explant (Fig. 5L–L″). These results together with our other data demonstrating limited amount of mitoses relative to robust cell cycle re-entry demonstrate restricted proliferative plasticity of adult supporting cells.

### Induction of DNA damage and cell cycle arrest in response to ectopic cD1 expression

**Figure 6:**
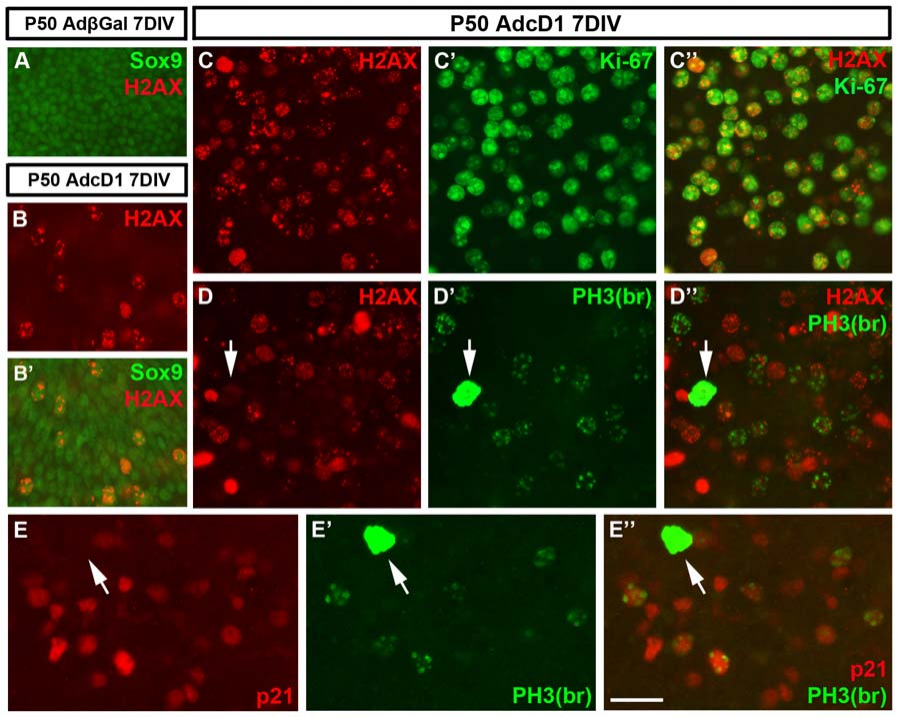
Induction of DNA damage in cell cycle reactivated supporting cells of the adult utricle. Absence of DNA damage and p21^Cip1^ expression in mitotic supporting cells. (A) Sox9+ supporting cells in AdβGal-infected explants do not express the DNA damage marker p-H2AX. (B,B') In contrast, several Sox9+/p-H2AX+ supporting cells are present at 7 DIV in explants transduced by AdcD1. (C-C″) Double-immunofluorescence shows that p-H2AX expression is restricted to Ki-67+ cells. (D-D″) p-H2AX is expressed in late G2 cells, based on the staining pattern obtained with the “PH3-Ser10 broad antibody” (see text). Observe that cells with PH3+ mitotic profiles lack p-H2AX foci (arrows in D',D″). (E-E″) In AdcD1-infected explants at 7 DIV, p21^Cip1^ is expressed in late G2 cells, based on double-staining with the “PH3-Ser10 broad antibody”. Note that PH3+ mitotic profiles do not show p21^Cip1^ staining (arrows in E',E″). Abbreviations: βGal, β-galactosidase; cD1, cyclin D1; PH3(na), phosphorylated histone H3-Ser10 narrow; PH3(br), phosphorylated histone H3-Ser10 broad; H2AX, phosphorylated histone H2AX. Scale bar, shown in E″: A-B', 40 µm; C-E″, 30 µm.

We continued by asking whether the prominent G2/M arrest is associated with DNA damage and activation of checkpoint mechanisms. In the case of the inner ear hair cells, unscheduled S phase entry triggers DNA damage in the form of DNA double-strand breaks (DSBs), leading to activation of the DNA damage response pathway and p53-mediated apoptosis [4], [6]. Activation (phosphorylation) of histone H2AX triggers recruitment of repair and checkpoint protein complexes to the replication break sites, and antibodies against phosphorylated histone H2AX (p-H2AX, Ser139) can be used to mark DSBs [31]. At 7 DIV, in contrast to AdβGal-infected explants, utricles transduced by AdcD1 showed high numbers of Sox9+ supporting cell nuclei with punctate p-H2AX staining, typical to the foci of DSBs (Fig. 6A-B′). This staining was specifically localized to Ki-67+ cells (Fig. 6C–C″). Taken together, cell cycle reactivation of supporting cells triggers DNA damage and activation of checkpoint mechanisms, leading to cell cycle arrest.

Interestingly, we found that the minor population of AdcD1-infected supporting cells that progressed into mitosis (0.6% of the amount of Ki-67+ supporting cells; see above) lacked or showed only a few p-H2AX foci, as evidenced by their PH3+/p-H2AX- nuclei (Fig. 6D-D″). Additionally, mitotic supporting cells were negative for p21^Cip1^ (Fig. 6E-E″). These results suggest that a part of cell cycle reactivated supporting cells may have the capacity for DNA repair, allowing progression through the G2/M boundary. Another possibility is that these cells experienced only slight DNA damage, allowing escape from checkpoint activation.

### p21^Cip1^ inactivation increases mitotic activity, but does not prevent cell cycle arrest of the main part of supporting cells with ectopic cD1 expression

We next studied the role of p21^Cip1^ in the observed G2/M arrest. p21^Cip1^ upregulation was seen already in replicating supporting cells, based on experiments using EdU as a S phase marker (data not shown). However, p21^Cip1^ induction might also be associated with G2 checkpoint activation and cell cycle arrest, based on the data showing that p21^Cip1^ is required for sustained G2 arrest after DNA damage, in a p53-dependent or p53-independent manner [32], [33]. At 7 DIV, similar to wild type specimens transduced by AdcD1, AdcD1-infected utricles from *p21^Cip1^* knock out mice showed high numbers of supporting cells with PH3+ profiles typical to the late G2 phase. In addition, supporting cells with PH3+ mitotic profiles were found (data not shown). Upon quantification, 12.8±2.6 mitotic supporting cells were found per mutant explant, which is 1.9% of the Ki-67+ supporting cell population (n = 6 explants). This value is significantly higher as compared to wild type explants (0.6%) (*P*<0.05). However, as widespread G2/M arrest persisted in the utricles from *p21^Cip1^* -/- mice as well, we conclude that p21^Cip1^ is an important, but not the sole factor mediating mitotic block of AdcD1-infected supporting cells.

### Supporting cells with ectopic cD1 expression lack signs of apoptosis

**Figure 7:**
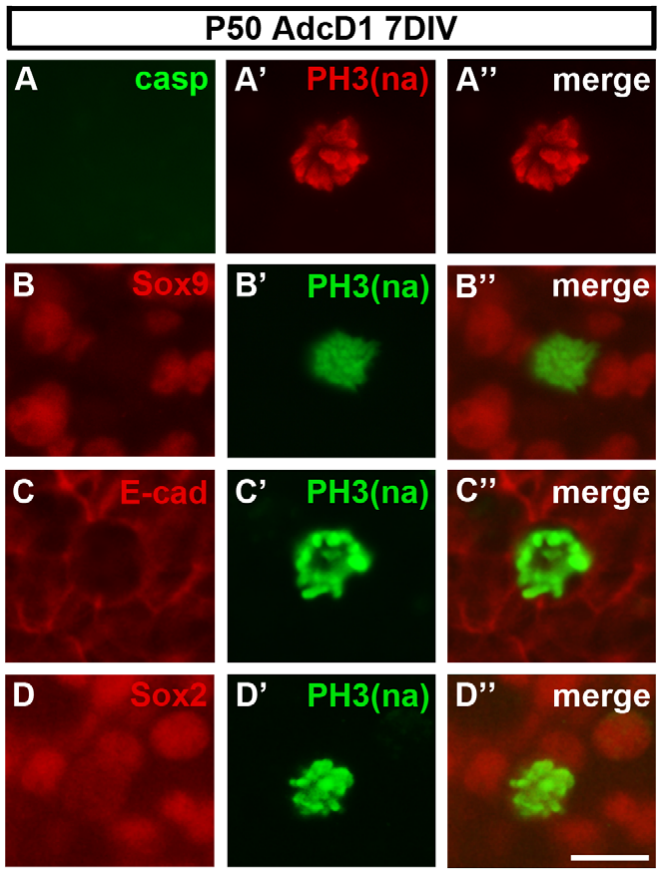
Lack of signs of apoptosis in mitotic supporting cells. Expression of supporting cell/epithelial markers in mitotic supporting cells. (A-A″) In AdcD1-infected explants at 7 DIV, supporting cells with PH3+ (“PH3-Ser10 narrow antibody”, see text) mitotic profiles are negative for cleaved caspase-3. (B-B″) Double-staining shows that PH3+ mitotic cells lack expression of the supporting cell marker Sox9. (C-C″) Double-staining shows that PH3+ mitotic cells are positive for E-cadherin. (D-D″) Double-staining shows that PH3+ mitotic cells are positive for the supporting cell marker Sox2. Abbreviations: cD1, cyclin D1; PH3(na), phosphorylated histone H3-Ser10 narrow; casp, cleaved caspase-3; E-cad, E-cadherin. Scale bar, shown in D″: A–D″, 10 µm.

To determine whether cell cycle reactivation of supporting cells is associated with apoptosis, paraffin sections and whole mounts from AcD1-infected P50 utricles were labeled by the ApopTag Kit (n = 8 explants) or immunostained for cleaved caspase-3 (n = 8 explants) at 7 and 12 DIV. We did not find apoptotic supporting cells (data not shown), despite the fact that our method of cell cycle reactivation triggered widespread cell cycle re-entry. These data suggest that, during the 12-day-long culture period, apoptosis was an infrequent event within the large population of DNA damaged and G2/M arrested supporting cells. Similarly, double-labeling experiments showed that PH3+ mitotic supporting cells were negative for cleaved caspase-3 (Fig. 7A-A″). However, it is difficult to detect apoptotic cells, because apoptosis is a rapid process and may occur in a scattered manner throughout the study period. Thus, low-level apoptosis might have been overlooked in our experiments.

### Sox9, but not Sox2 expression is altered in mitotic supporting cells

We next investigated whether the expression of lineage-specific transcription factors is altered in cell cycle reactivated supporting cells. Of the few transcription factors thus far localized to mature utricular supporting cells, we focused on the HMG-box factors Sox9, the cell-type-specific marker used in the present study, and Sox2 [34]. Intriguingly, Sox9 expression was dynamically changed along cell cycle progression. It was found in all Ki-67+ supporting cells, except for those that had mitotic profiles. This was confirmed by the Sox9-/PH3+ status of these cells (Fig. 7B-B″). PH3+ cells showed E-cadherin immunoreactivity, confirming their epithelial identity (Fig. 7C-C″). Further, in triple-labeling experiments, we confirmed that Sox9-/PH3+ cells were not hair cells, based on the lack of parvalbumin expression (data not shown), Interestingly, in contrast to Sox9, expression of Sox2 was maintained in mitotic supporting cells (Fig. 7D-D″). Together, these results show that progession of supporting cells into mitosis is associated with selective changes in their transcriptional program.

### Proliferative plasticity of early postnatal supporting cells with ectopic cD1 expression

**Figure 8:**
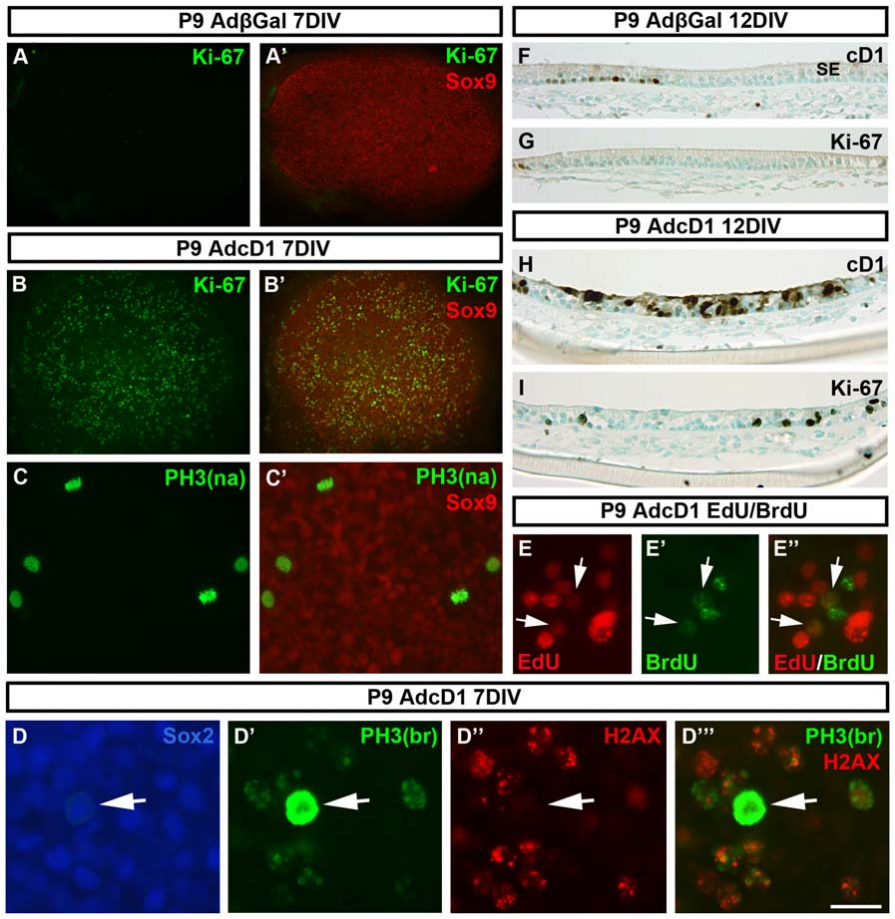
Cell cycle progression of early postnatal utricular supporting cells in response to ectopic cD1 expression. (A,A') An AdβGal-infected P9 explant maintained for 7 DIV and therafter double-stained for Sox9 and Ki-67 shows the absence of proliferating supporting cells. (B,B') Ki-67/Sox9 double-immunofluorescence shows high proliferative activity of supporting cells in AdcD1-infected P9 explants. (C,C') An AdcD1-infected explant shows supporting cells with PH3+ mitotic profiles (PH3-Ser10 narrow antibody”, see text). As compared to adult explants, the amount of mitotic cells is higher at this age (compare to Fig. 5E-E″). (D-D’’’) Triple-immunofluorescence shows several Sox2+ supporting cells that have progressed into late G2 phase, based on dotted pattern of PH3 labeling (PH3-Ser10 broad antibody”, see text). p-H2AX reactivity indicates that these cells are DNA damaged. Note that the cells with PH3+ mitotic figures lack p-H2AX foci (arrows in D-D’’’). (E-E″) Labeling for the thymidine analogs EdU and BrdU (see text for pulsing regimen) shows several supporting cell nuclei positive for one of the analogs. Additionally, a few double-positive nuclei can be seen (arrows). (F,G) Adjacent histological sections through an AdβGal-infected explant at 12 DIV show a small number of supporting cells with endogenous cD1 expression (F) and the lack of Ki-67+ cells (G). (H) As shown at 12 DIV, ectopic cD1 accumulates both in the nucleus and cytoplasm of supporting cells of AdcD1-infected explants, similar to adult specimens. (I) An adjacent section shows that Ki-67 expression is maintained in these long term cultures. Observe the lack of hyperplasia of AdcD1-infected P9 sensory epithelia. Abbreviations: cD1, cyclin D1; PH3(na), phosphorylated histone H3-Ser10 narrow; PH3(br), phosphorylated histone H3-Ser10 broad; H2AX, phosphorylated histone H2AX; EdU, 5-ethynyl-2′-deoxyuridine; BrdU, bromodeoxyuridine; SE, sensory epithelium. Scale bar, shown in E″: A–B', 140 µm; C,C', 50 µm; D-D’’’, 30 µm; E-E″, 40 µm; F–I, 70 µm.

**Figure 9:**
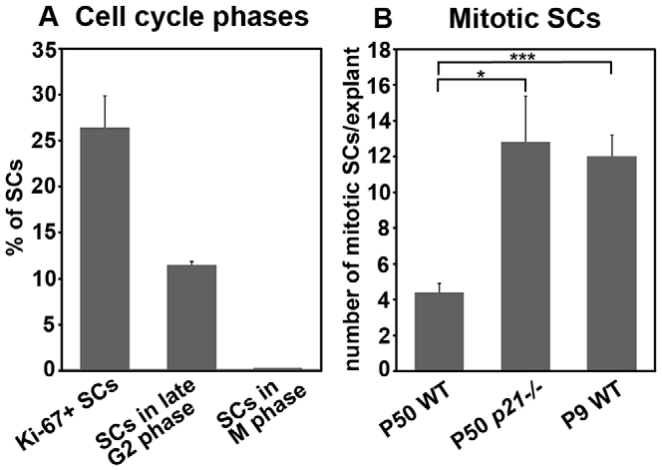
Frequency of supporting cells at different cell cycle phases in AdcD1-infected utricular explants. (A) The percentages of Ki-67+ supporting cells (26.4%±3.5, n = 7 explants, a total of 11276 cells counted), supporting cells undergoing late G2 phase (11.5%±0.4, n = 7, a total of 8679 cells counted) and mitotic supporting cells (0.16%±0.02, n = 8) of the total number of these cells in AdcD1-infected explants at 7 DIV. Mean ± SEM. (B) The actual number of mitotic supporting cells per AdcD1-infected utricle at 7 DIV. Wild type P50 utricles (4.4±0.5, , n = 8 explants), *p21^Cip1^*-/- P50 utricles (12.8±2.6, n = 6), wild type P9 utricles (12±1.2, n = 9) are compared. Statistical significance: *, *P*<0.05; ***, *P*<0.001. Abbreviation: SCs, supporting cells.

Finally, we determined whether the observed limitations in cell cycle progression are age-dependent. We found that the response of P9 explants to ectopic cD1 expression largely corresponded to the response of adult specimens. At 7 DIV, AdcD1-, but not AdβGal-infected P9 supporting cells displayed widespread cell cycle re-entry (Ki-67; Fig. 8A–B′). Quantification at 7 DIV revealed the presence of 12±1.2 cells with PH3+ mitotic profiles per P9 explant (n = 9), which is 2,5% of the amount of Ki-67+ supporting cells in these explants. This mitotic activity is significantly higher relative to the corresponding value in P50 explants (0.6%) (*P*<0.001). Similar to adult utricles, mitotic profiles largely represented prophase or metaphase (Fig. 8C-C′). Despite increased mitotic activity, labeling with the “PH3-Ser10 broad antibody” showed high numbers of G2/M arrested supporting cells, and these cells clearly predominated over mitotic cells. G2/M arrested supporting cells were DNA damaged, based on the presence of p-H2AX foci (Fig. 8D-D′′′). Analogous to adult explants, mitotic supporting cells in P9 utricles lacked or showed very weak p-H2AX staining. These cells were negative for Sox9, but positive for Sox2 (Fig. 8D-D′′′). Experiments with the thymidine analogs EdU and BrdU, using the same pulsing paradigm as with adult explants, revealed the presence of EdU+/BrdU+ supporting cells, indicating that these cells had replicated twice (Fig 8E-E″). Similar to adult explants, only rare double-positive supporting cells were found. This is consistent with the lack of hyperplasia of the sensory epithelium, as analyzed in histological sections at 12 DIV (Fig. 8F-I). Further, early postnatal explants showed accumulation of ectopic cD1 in the cytoplasm of supporting cells, similar to adult explants (Fig. 8H). Collectively, comparison of P9 and P50 utricular supporting cells forced into the cell cycle shows that mitotic progression is more plastic in the younger age group. However, marked restrictions in proliferative potential can be seen at this age as well. Fig. 9 summarizes these data.

## Discussion

This study was undertaken to penetrate into the proliferative potential of adult inner ear supporting cells. We ectopically expressed cD1 in utricular supporting cells in explant cultures and studied the capacity of these normally quiescent cells to progress through the cell cycle and divide. Most of earlier studies on cell cycle regulation in the inner ear supporting cells have analyzed immature, rather than fully differentiated cells. Furthermore, prior studies on proliferative capacity of supporting cells have largely focused on initial events of the cell cycle, i.e. on the ability to resume DNA synthesis, and only little attention has been paid to the capacity of these cells to progress through later phases of the cell cycle. The current results reveal that DNA damage-induced cell cycle arrest is a prominent consequence of cell cycle re-entry of mature supporting cells. We discuss below the implications of these findings for triggering therapeutically relevant expansion of the supporting cell pool. Utricular (vestibular) and cochlear supporting cells share developmental history and have several similarities in their phenotypic characteristics. Therefore, although cochlear supporting cells show higher structural complexity, data on proliferative potential of mature utricular supporting cells are likely to be relevant when evaluating this potential in the inner ear in general.

Adenoviruses infect postnatal inner ear supporting cells with high efficiency, as demonstrated in the present study using AdβGal and AdcD1 vectors. AdcD1 infection triggered widespread cell cycle re-entry of supporting cells, as evidenced by Ki-67 induction. However, Ki-67+ cells represented only a part of the total population of AdcD1-infected supporting cells. One likely reason for the lack of a proliferative response in all infected supporting cells is that ectopic cD1 distinctly accumulated in the cytoplasm. Exogenous cD1 has also been shown to accumulate in the cytoplasm of quiescent cardiomyocytes and neurons, leading to impaired phosphorylation of nuclear pRb and, thus, to impaired proliferation [35]–[37]. In these prior studies, subcellular distribution of ectopic cD1 was linked to developmental plasticity: embryonic, still proliferating cardiomyocytes showed nuclear expression, while neonatal, quiescent cardiomyocytes sequestered cD1 in the cytoplasm [35], [37]. In those studies, ectopic cD1 was largely restricted to the cytoplasm, in contrast to our study in which ectopic cD1 was expressed both in the cytoplasm and nucleus of supporting cells. In any case, the partially impaired translocation of cD1 into the nucleus may impair the mitogen responsiveness of utricular supporting cells. Thus, cytoplasmic cD1 sequestration may be one of the mechanisms underlying the maintenance of the postmitotic state of supporting cells. Further, it may be an important barrier in the mitogen-based attempts to stimulate therapeutically relevant proliferation of supporting cells.

Cell cycle reactivated supporting cells showed prominent changes in the expression of the polypeptides of the Cip/Kip family of CKIs. p27^Kip1^ is the predominant CKI in the inner ear supporting cells [14], [15], [23]. We found marked p27^Kip1^ downregulation in utricles transduced by AdcD1 and, specifically, this downregulation was localized to Ki-67+ supporting cells. p27^Kip1^ suppression likely facilitates cell cycle re-entry. Of interest, an earlier study earlier has shown that dissociated supporting cells from the neonatal mouse cochlea can re-enter the cell cycle and this event was linked with p27^Kip1^ downregulation [22]. It was further shown that p27^Kip1^ levels are unaltered in supporting cells sorted out from more mature cochleas, in line with the inability of these cells to re-enter the cell cycle [22]. Combined with our results, it seems that adult utricular supporting cells are more plastic in their response to cell cycle reactivation as compared to mature cochlear supporting cells, and this may in part depend on differential plasticity at the level of p27^Kip1^ protein expression.

Utricular supporting cells normally show hardly detectable p21^Cip1^ expression [2], [23], a result that was confirmed in control cultures of the current study. We found distinct upregulation of both the p21^Cip1^ protein and mRNA in supporting cells transduced by AdcD1. This upregulation was seen already during DNA replication, suggesting that p21^Cip1^ induction is a rate-limiting step in cell cycle re-entry. However, the extent of proliferative activity, as assessed by Ki-67 staining, was comparable in explants from the *p21^Cip1^*knock out and wild type mice, excluding a primary role for p21^Cip1^ in S phase activity. This conclusion is consistent with earlier data showing a non-proliferative status of the inner ear supporting cells from the *p21^Cip 1^*knock out mice [2]. Although we could not obtain direct evidence for the role of p21^Cip1^ at the early phases of cell cycle reactivation, it might play a collaborative role with p27^Kip1^, similar to p21^Cip1^ and the Ink4 family CKI p19^Ink4d^ in cochlear hair cells [4]. Our results more clearly point to the role of p21^Cip1^ in limiting supporting cell mitoses. This is consistent with the known function of p21^Cip1^ as a transcriptional target of p53. However, as only a part of cell cycle reactivated supporting cells from the *p21^Cip1^* knock out mice were relieved from proliferative arrest, additional regulators seem to be involved.

The most striking finding of our study is the failure of the main part of AdcD1-infected utricular supporting cells to traverse the G2/M boundary. This failure has not been shown in prior studies in which the cell cycle machinery of neonatal or adult inner ear supporting cells has been manipulated. The fact that adult, normally quiescent hepatocytes display robust M phase entry in response to ectopic cD1 expression [38] supports the notion that deregulated cD1 expression itself is not the primary cause of the observed G2 arrest. We found that mitotic activity was higher in P9 than in adult supporting cells. However, also these younger cells showed infrequent mitoses relative to robust cell cycle re-entry and the G2/M block was a predominant event. Chromatin condensation, a hallmark of mitosis, is initiated in the late G2 phase [29], [30]. One of the antibodies against PH3-Ser10 used in the current study reacted with the prominent chromatin condensation of mitotic cells. This antibody showed only a few mitotic profiles per AdcD1-infected explant. The other PH3-Ser10 antibody additionally labeled the weak chromatin condensation present at the late G2 phase and G2/M transition, apparently due to its higher sensitivity. This antibody reacted with a large number of supporting cells in AdcD1-infected utricles, showing a weak, dotted labeling pattern, typical to early chromatin condensation. We localized p-H2AX foci to these cells, suggesting that DNA damage, likely linked with aberrant DNA replication (replication stress; 39), triggers activation of checkpoint mechanisms, leading to cell cycle arrest. Similarly, DNA damage and/or G2/M arrest have been demonstrated in some other quiescent cell types in response to manipulation of the cell cycle [6], [8], [37], [40]–[42]. In contrast to these arrested cells, some other cell types, such as cochlear outer hair cells and many neuronal populations, are hypersensitive to forced proliferation and die rapidly after S phase initiation [6], [7]. Based on the current experiments, we can only speculate about the eventual fate of G2/M arrested utricular supporting cells. The facts that these cells were DNA damaged and thus genomically instable and that differentiated, postmitotic cells in general have a poor DNA repair capacity suggest for impaired survival and delayed death, rather than indefinite growth arrest.

Although the majority of AdcD1-infected supporting cells were arrested at the G2/M boundary, a small fraction of them progressed into mitosis. Further, we found rare supporting cells double-positive for the thymidine analogs EdU and BrdU, suggesting that these cells had completed a cell cycle and continued with another replication. These observations speak for proliferative potential of a part of supporting cells following forced cell cycle re-entry *in vitro*. However, we cannot exclude the possibility that the EdU+/BrdU+ cells represented G2 arrested supporting cells that underwent endoreduplication, resulting in polyploidy [33]. The infrequency of EdU+/BrdU+ cells is in accordance with the fact that the sensory epithelium did not show hyperplasia. These results are in accordance with the general notion that adult mammalian postmitotic cells lack proliferative potential. However, it should be noted that recent data on certain cell types, e.g. mammalian cardiomyocytes, have challenged this notion and demonstrated complete cell divisions and clonal expansion in specific experimental settings [43]–[47]. Importantly, in some of these studies, forced cell cycle re-entry was linked with dedifferentiation, analogous to the natural regenerative events in non-mammalian species. Dedifferentiation in mammals has been shown to be required not only for S phase re-entry, but also for progression into mitosis [43], [46], [47]. In this respect, the changes that we observed in AdcD1-infected utricles in the expression of Sox9, a transcription factor normally present in all inner ear supporting cells, were interesting. Sox9 was present in replicating and G2/M arrested supporting cells, but absent from mitotic cells. However, as Sox2 expression was maintained in mitotic supporting cells, the current data alone do not allow us to suggest that Sox9 suppression reflects cellular dedifferentiation. Based on the data that Sox9 promotes survival of other types of differentiated cells, such as chondrocytes [48], another possibility is that its suppression reflects a stressful, unstable condition and that mitotic catastrophe-linked cell death may be a consequence of forced proliferation. We did not obtain evidence of death of supporting cells undergoing mitosis, but the fact that most mitotic figures represented early, rather late stages of M phase supports this possibility.

### Conclusions

The inner ear supporting cells of birds, fishes and reptiles can produce daughter cells, an event that together with the capacity for phenotypic conversion into hair cells underlies the natural regenerative capacity. The present study shows that the normally postmitotic supporting cells of the mouse utricle can re-enter the cell cycle in response to ectopic expression of cD1, similar to inducible deletion of *p27^Kip1^* [13]. Our data demonstrate that after this initial cell cycle reactivation there are considerable obstacles in cell cycle progression. It is likely that these obstacles should be overcome if effective and harmless therapeutic interventions are realized. One barrier is the ineffective import of cD1 into nucleus, which may be a problem in conjunction with the use of exogenous mitogens. The other, critical barrier is DNA damage-associated cell cycle arrest, implying the need for interventions that target genes promoting DNA repair and G2/M transition. Concerning the small population of supporting cells that showed the capacity for M phase entry, we cannot exclude the possibility that persistent cD1 overexpression induces abnormalities in the mitotic apparatus, as earlier demonstrated both in malignant and normal cells [49], and that these alterations impair cellular survival and regrowth. Of note, also *Rb*-inactivated cells show mitotic defects, such as centrosome overduplication and chromosome instability [50], [51]. Based on the current data underscoring barriers in cell-cycle activity in the mouse utricular supporting cells, dedifferentiation might be a process to be taken into forefront. Dedifferentiation is an integral part of proliferative plasticity in non-mammalian species. Recent data show that this is also the case also with certain mammalian cell types that have been forced into the cell cycle and that have successfully divided [44], [47], [48]. Thus, manipulations aimed at reverting mature supporting cells back to a less-differentiated stage might confer proliferative plasticity, which, optimally, might be followed by redifferentiation and transdifferentiation, leading to preservation of cell numbers and to replacement of lost sensory hair cells.

## Materials and Methods

### Mice

Inner ear explant cultures were prepared from the NMRI (control) and *p21^Cip1^* knock-out mice strains. *p21^Cip1^* knock out mice have been described previously [52; obtained from Jackson Laboratory]. Genotyping by PCR using DNA extracts from tails was performed as previously described [52]. The day of birth was considered as P0. All animal work has been conducted according to relevant national and international guidelines. Approval has been obtained from the Finnish Committee of Experimental Animal Research.

### Explant cultures and viral infections

Organotypic cultures of the utricular sensory epithelium were established at P50 and P9. Explants were maintained on pieces of Nuclepore filter membrane (Whatman) placed on a metal grid in Dulbecco's modified Eagle's medium/F-12 medium supplied with 2 mM L-glutamine and penicillin (100 U/mL) (Gibco/Invitrogen) and 10% fetal bovine serum (FBS) (HyClone/Thermo Scientific). Incubations were done in a humidified 5% CO_2_ atmosphere at 37°C.

Adenoviral (serotype 5) vectors harbouring the *βGal* and c*D1* transgenes were used. Both transgenes were under the *CMV* promoter. Cloning and propagation of these recombinant viruses have been described previously [53]. Prior to infection, explants were stabilized on filters for 12 h. Explants were infected for 6 to 8 h with AdβGal at the concentration of 2,0×10^7^ pfu/ml or with AdcD1 at the concentration of 2,3×10^7^ pfu/ml. Infections were done in 25 µl drops of medium containing 2% FBS. Thereafter, explants were maintained for 2 to12 DIV in medium containing 10% FBS. Medium was changed every other day.

### Whole mount specimens

Explants were fixed with 4% paraformaldehyde (PFA) in phosphate buffered saline for 15 min. Double- and triple-immunofluorescent stainings were performed using the following antibodies: goat polyclonal parvalbumin (Swant, #PVG-214, 1∶2000); mouse monoclonal β-Galactosidase (Promega, #Z378A, 1∶250); rabbit monoclonal cD1 (LabVision/Thermo Scientific, #RM-9104, 1∶250); rabbit monoclonal Ki-67 (LabVision/Thermo Scientific, #RM-9106, 1∶250); mouse monoclonal Ki-67 (Novocastra Laboratories, #NCL-Ki67-MM1, clone MM1, 1∶100); mouse monoclonal PH3-Ser10 (Cell Signaling Technology, #9706, clone 6G3, referred in the text as PH3-Ser10 narrow antibody, 1∶250); rabbit polyclonal PH3-Ser10 (Cell Signaling Technology, #9701, referred in the text as PH3-Ser10 broad antibody, 1∶250); rabbit monoclonal cleaved caspase-3 (Cell Signaling Technology, #9664, 1∶250); mouse monoclonal p21^Cip1^ (BD Biosciences, #556431, clone SXM30, 1∶100); mouse monoclonal p27^Kip2^ (BD Biosciences, #610241, clone 57, 1∶2000); rabbit polyclonal Sox9 (Millipore, #AB5535, 1∶2000); mouse monoclonal p-H2AX (Millipore, #05-636, clone JBW301, 1∶250); goat polyclonal Sox2 (Santa Cruz Biotechnology, #SC-17320, 1∶2000) and rat monoclonal E-cadherin (Sigma, #U3254, 1∶500). Secondary antibodies conjugated to Alexa 350, Alexa 488 and Alexa 594 were used for visualization and ProLong Gold anti-fade reagent (Molecular Probes/Invitrogen) for mounting. To detect apoptotic cells, whole mount specimens were stained with the ApopTag Plus Fluorescence *In Situ* Apoptosis Detection Kit (Millipore) according to manufacturer's instructions.

### Labeling with thymidine analogs

Utricular explants were pulse-labeled first with 10 µM BrdU (GE Healthcare) for 24 h between days 3 and 4. A second pulse with 10 µM EdU (Molecular Probes/Invitrogen) was given for 24 h between days 7 and 8. Explants were fixed with PFA at 9 DIV. Specimens were first stained with the Click-iT EdU Imaging Kit (Invitrogen) according to manufacturer's instructions. Thereafter specimens were treated with 2 M HCl for 20 min before staining with the mouse monoclonal BrdU antibody (LabVision/Thermo Scientific, Ab-2, #MS-949-P, clone BRD.2, 1∶1000). A part of explants were pulsed only with EdU between days 6 and 7, and fixed immediately thereafter.

### Histological sections

A part of explants were embedded into paraffin and cut to 5-µm-thick sections. Sections were stained with the rabbit monoclonal Ki-67, rabbit monoclonal cD1, rabbit monoclonal PH3 and rabbit monoclonal cleaved caspase-3 antibodies. Epitopes were unmasked by microwave heating (800 W) in 10 mM citrate buffer, pH 6.0, for 10 min. Detection was done with Vectastain Elite ABC kit and DAB Detection kit (Vector Laboratories). Sections were counterstained with 3% methyl green and mounted in Permount (Fisher Scientific). In addition, paraffin sections stained with hematoxylin (Shandon Instant Hematoxylin, Thermo Scientific) only were analyzed. For semithin sections, explants were fixed for 15 min in 2.5% glutaraldehyde in 0.1 M phosphate buffer, pH 7.4. Specimens were postfixed in 1% osmium tetroxide and embedded in Epon (Electron Microscopy Sciences). Sections were cut at 0.5 µm and stained with 2% toluidine blue.

*In situ* hybridization was performed with ^35^S-labelled riboprobes on PFA-fixed and paraffin-embedded sections as described [54]. Utricular explants were analyzed for *p27^Kip1^* and *p21^Cip1^* expression. Sections were counterstained with hematoxylin. Images were processed using Adobe Photoshop CS4 (Adobe Systems). Autoradiographic silver grains in the dark field image were selected, colored red and superimposed onto the brightfield image.

Wholemounts and histological sections were analyzed with a BX61 microscope (Olympus) using bright- and darkfield optics and epifluorescence. Images were acquired through the CCD colour camera (DP70) and the cell∧F software (Olympus), and processed using Adobe Photoshop CS4 (Adobe Systems).

### Quantification

For quantification of hair cell loss, numbers of parvalbumin+ hair cells were counted in AdβGal -infected explants at 7 DIV. When counting this cellular loss, the total number of 3246 hair cells in the adult mouse utricle, as earlier determined [55], was used as a baseline. Total number of supporting cells is 2754 per adult utricle [55]. For quantification of proliferative activity in AdcD1-infected explants, Ki-67+/Sox9+ supporting cells were counted from two 20x microscopic fields per explant. Numbers of supporting cell undergoing late G2 phase (“PH3-Ser10 broad antibody”) were counted from similar fields. For quantification of mitotic activity, the total number of PH3+ mitotic profiles per explant was counted. Data are shown as averages with standard error of the mean. The two-tailed Student's *t* test was used to analyze differences between two groups. Values were regarded significant at *P*<0.05.
